# Cleaning efficacy of EDDY versus ultrasonically-activated irrigation in root canals: a systematic review and meta-analysis

**DOI:** 10.1186/s12903-023-02875-6

**Published:** 2023-03-17

**Authors:** Xiaojun Chu, Shuting Feng, Weiqing Zhou, Shuaimei Xu, Xiongqun Zeng

**Affiliations:** Department of Endodontics, Stomatological Hospital, School of Stomatology, Southern Medical University, No 366 Jiangnan Avenue South, Guangzhou, 510280 Guangdong China

**Keywords:** Irrigation, Ultrasonic activation, EDDY, Systematic review

## Abstract

**Background:**

Ultrasonically-activated irrigation (UAI) is effective in root canal irrigation but may damage canal walls. EDDY is a sonic activation system with flexible working tips that cause no harm to dentinal walls. This review explores the intracanal cleaning efficacy of EDDY compared with UAI in vitro.

**Methods:**

The systematic review was registered in the PROSPERO database (CRD42021235826). A literature search was conducted in six electronic databases. In vitro studies that compared the removal of smear layer, debris, soft tissue or microbes in root canals between EDDY and UAI were included. Data extraction and quality assessment were performed. Meta-analyses were conducted on smear layer removal and debris elimination with the standardized mean difference (SMD). Heterogeneity was measured using the I^2^ test and the Chi^2^ test. The random-effect model was used when I^2^ > 50%, or *p* < 0.1, otherwise the fixed-effect model was applied. The level of significance was set at *p* < 0.05.

**Results:**

19 articles were included in this systematic review and 7 articles were included in meta-analyses. Meta-analyses on smear layer removal showed unimportant differences between EDDY and UAI at any canal third (coronal [SMD = 0.08, 95% confidence interval (95%CI): -0.29 to 0.45; *p* = 0.44, I^2^ = 0%]; middle [SMD = 0.02, 95% CI: -0.44 to 0.47; *p* = 0.94, I^2^ = 0%]; apical [SMD = 0.01, 95%CI: -0.35 to 0.38; *p *= 0.70, I^2^ = 0%]). Meta-analyses on debris removal evaluated by scanning electron microscope (coronal [SMD = 0.03, 95% CI: -0.41 to 0.46; *p* = 0.27, I^2^ = 23%]; middle [SMD = -0.24, 95% CI: -0.83 to 0.35; *p* = 0.80, I^2^ = 0%]; apical [SMD = 0.24, 95%CI: -0.20 to 0.67; *p* = 0.36, I^2^ = 2%]) and micro-CT (SMD = 0.36, 95% CI: -0.67 to 1.40; *p* = 0.03, I^2^ = 70%) both found insignificant differences. No meta-analysis was undertaken on soft-tissue removal and disinfection due to the various study designs, but the qualitative analyses implied that EDDY achieved similar performance to UAI in both aspects.

**Conclusions:**

Limited evidence indicated that EDDY was comparable to UAI in removing smear layer, debris, soft tissue and microbes ex vivo. Considering UAI may damage canal walls, EDDY might be a substitute for UAI in irrigation activation. But more randomized clinical trials are required to explore the clinical extrapolation of the results in this review.

## Background

Mechanical preparation in root canal treatment produces lots of smear layer and debris [1]. The presence of smear layer will hinder irrigants and medication from entering tubules for thorough disinfection [2, 3]. Besides, restricted by the conoid shape of files, canal walls of narrow anatomic sites will stay unprepared after instrumentation [4, 5], and the soft tissue and biofilms in these areas cannot be mechanically removed. Remaining debris and soft tissue shelters microbes [6, 7], which may leave long-term potential risks if not eradicated [8]. Therefore, root canal irrigation is indispensable for a thorough and effective root canal treatment as it can clean and disinfect the unprepared areas [9].

Needles and syringes with various vent designs are the traditional instruments to conduct irrigation. However, the intracanal cleaning efficacy of needle irrigation is unsatisfactory [10–13]. Agitation of irrigants is thus required to improve the irrigation effects. Ultrasonically-activated irrigation (UAI) is the most used endodontic activation device in both America [14] and the UK [15]. With a vibration frequency of 25,000 Hz to 40,000 Hz, UAI activates irrigation solution by generating a cavitation effect [16, 17] and acoustic microstreaming [17]. The ultrasonic oscillation also gives rise to circumferential shear stress acting on canal walls [18] and may assist in removing organic remnants and byproducts produced by instrumentation. However, ultrasonic activation may damage dentinal walls and develop microcracks [19–21] probably due to its alloy working tips [22] and high-frequency oscillation. Therefore, an airscaler-powered activation system (EDDY; VDW GbmH, Munich, Germany) driven at a frequency of 5000 to 6000 Hz has been introduced and studied. It uses polyamide tips with only one available size (20/02) that are much more flexible than stiff metal tips. According to the manufacturer, EDDY (ED) tips move in a three-dimensional way at a high amplitude. Many studies have been carried out to compare the cleaning effects between UAI and ED [23–25]. However, their results are contradictory even though the vibration frequencies of the two activation systems contrast sharply.

Therefore, in view of the above factors, a systematic review is in need to appraise the relevant studies to further explore the cleaning efficacy of ED in comparison to UAI. The focused question of this review is established on the PICO framework: Does UAI (I) achieve better performance on canal cleanliness including the elimination of smear layer, debris, soft tissue and bacterial (O) compared to ED (C) in vitro (P)?

## Methods

This systematic review followed the Preferred Reporting Items for Systematic Reviews and Meta-Analyses (2020) checklist and was registered in the PROSPERO database (CRD42021235826).

### Eligibility criteria

We included articles that studied UAI in comparison to ED on at least one of the following aspects: the disinfection effectiveness or the ability to remove the intracanal smear layer, debris or soft tissue. Experiments should be performed either with canal models or extracted mature permanent teeth without root canal treatment and fractures. Studies conducted on open canal systems were excluded.

### Search strategy

A systematic literature search was conducted on 7 February 2023 using the following electronic database: Pubmed, Embase, Web of Science, Cochrane Library, Scopus and SinoMed (http://www.sinomed.ac.cn/). The publication date was restricted to 2015 and beyond, as EDDY was first introduced in 2015 [26]. No filter on the language was set in the search process. Eight keywords (“root canal”, “irrigation”, “EDDY”, “ultrasonic”, “smear layer”, “debris”, “tissue” and “disinfection”) were selected as the primary search terms. When similar words or expressions came up, terms were enriched or amended and the search was repeated to retrieve maximum and accurate results. An example of the final search strategy and the corresponding results in Pubmed are listed in Table 1. The references of all included articles were searched as well to avoid missing information.

**Table 1:** Pubmed search strategy

Number	Search Strategy	Results
#1	(canal[Title/Abstract]) OR (intracanal[Title/Abstract])	85,738
#2	(irrigation[Title/Abstract]) OR (irrigate[Title/Abstract]) OR (irrigant[Title/Abstract]) OR (activation[Title/Abstract]) OR (activate[Title/Abstract]) OR (activated[Title/Abstract])	1,654,266
#3	(sonic[Title/Abstract]) OR (sonically[Title/Abstract]) OR (EDDY[Title/Abstract])	14,488
#4	(ultrasonic[Title/Abstract]) OR (ultrasonically[Title/Abstract])	59,054
#5	(smear layer[Title/Abstract]) OR (debris[Title/Abstract])	25,486
#6	(bacterial[Title/Abstract]) OR (bacteria[Title/Abstract]) OR (antibacterial[Title/Abstract]) OR (antibiofilm[Title/Abstract]) OR (biofilm[Title/Abstract]) OR (disinfection[Title/Abstract]) OR (microbe[Title/Abstract]) OR (microbial[Title/Abstract]) OR (Enterococcus faecalis[Title/Abstract]) OR (microorganism[Title/Abstract]) OR (microbiology[Title/Abstract]) OR (microbiota[Title/Abstract])	1,135,648
#7	(tissue[Title/Abstract]) OR (mucosa[Title/Abstract]) OR (meat[Title/Abstract]) OR (collagen[Title/Abstract]) OR (gelatin[Title/Abstract]) OR (gum[Title/Abstract]) OR (gel[Title/Abstract]) OR (hydrogel[Title/Abstract])	2,271,777
#8	#5 OR #6 OR #7	3,317,882
#9	(#1) AND (#2) AND (#3) AND (#4) AND (#8)	86
#10	(#1) AND (#2) AND (#3) AND (#4) AND (#8) Filters: from 2015—2023	62

### Study selection

Two reviewers (CX and FS) screened the titles and abstracts of all the articles independently after the exclusion of duplicate records. When either reviewer found a study potentially eligible according to the inclusion criteria, the full text was obtained. Both reviewers assessed the full texts and decided on final inclusion by consensus or in consultation with a third reviewer (ZX).

### Data extraction

Tables for data extraction were designed according to the following items: author(year), sample size and types, apical preparation size, parameters of activation systems, irrigation protocols, research indications, observational sites, evaluation methods and main results. Two reviewers (CX and FS) performed the data extraction independently. Study authors would be contacted for clarification if any uncertainty came up. Disagreements were resolved through discussion or with the help of a third reviewer (ZX).

### Quality assessment

The assessment of the included studies was conducted based on the method used in previous systematic reviews [27, 28] with adjustments. The assessment items were as follows: (i) sample size calculation, (ii) samples with similar dimensions, (iii) standardization of procedures, (iv) blinding of sampling and assessment, (v) statistical analysis and (vi) other bias. Each item of each included study was judged as “low” (green dot) or “high” (red dot) risk of bias. Two trained reviewers (CX and ZW) performed the assessment independently. Disagreements were resolved by discussion or with the help of a third reviewer (ZX). Each included study was given an overall judgment according to the risk of bias in each domain:

- Low risk of bias: studies that had all the items with low risk of bias;
- Moderate risk of bias: studies that had 4 to 5 items with low risk of bias;
- High risk of bias: studies that had less than 4 items with low risk of bias.

### Meta-analysis

Qualitative analyses were performed separately on the reduction of smear layer, debris, soft tissue and microbes. Meta-analyses were conducted only when the required data were accessible and the analyses were meaningful; that is, if the study designs and outcome variables were similar enough for the quantitative synthesis to make sense. Therefore, only the articles evaluating the smear layer removal and the debris elimination with available data were included in the respective meta-analyses, because they shared similar methodologies. Standardized mean difference (SMD) and the corresponding 95% confidence interval (95% CI) were calculated for each eligible study. Heterogeneity was measured by the Chi^2^ test and the I^2^ test. When *p* < 0.1 or I^2^ > 50%, heterogeneity was regarded as substantial and the random-effects model was used to estimate the overall effect size, otherwise the fixed-effect model was used. Statistical heterogeneity was explored by analyzing methodological diversity rather than subgroup analyses or meta-regression, on account of the inadequate number of studies in each meta-analysis. All analyses were performed using Review Manager software (Revman 5.4.1).

## Results

### Study selection

**Fig. 1:**
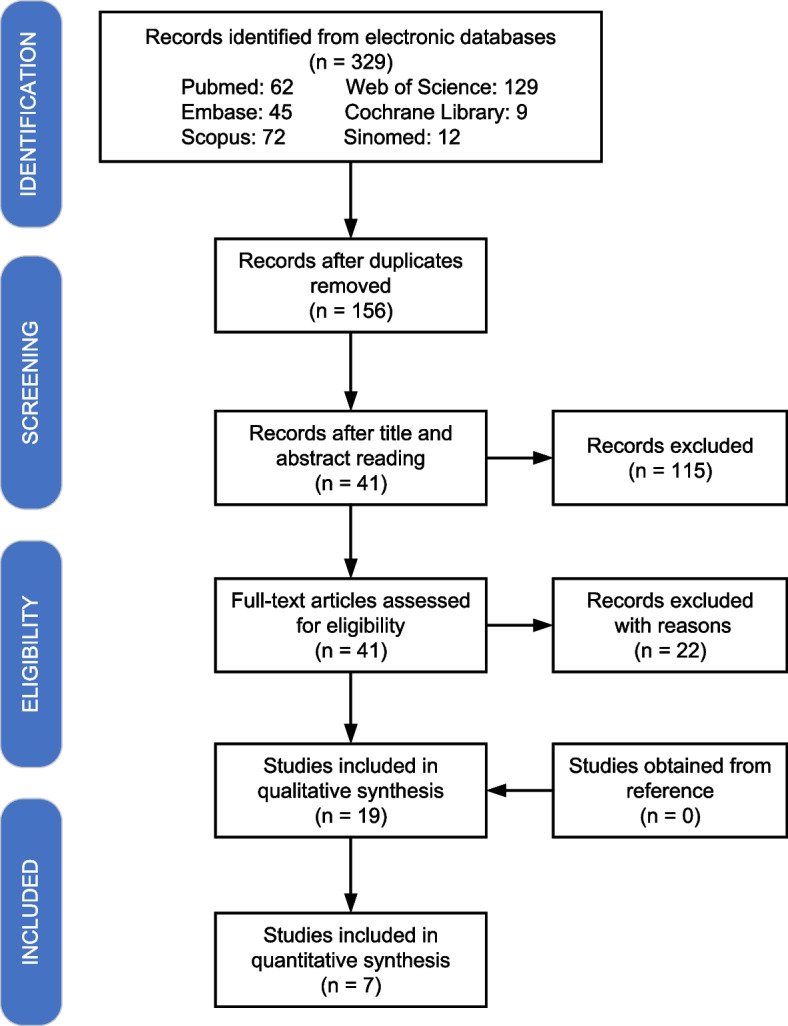
A flowchart of the literature search process

The search process was shown in Fig. 1. The database search resulted in 329 records. 173 duplicates and 115 ineligible records were removed after titles and abstracts were screened, remaining 41 records for full-text evaluation. After assessing all the full texts in detail, we excluded 12 articles [12, 29–39] as they did not study EDDY and other 10 articles [40–49] as apices were not sealed before root canal irrigation. 19 studies [13, 22–25, 50–63] meeting all the inclusion criteria were included in the review. No additional study was added after a manual search of the reference of the included articles.

### Characteristics of the included studies

The characteristics of all the 19 included studies are listed in Tables 2 and 3. Five articles [50, 54, 57, 60, 63] compared the removal of the smear layer between EDDY and UAI. The elimination of debris was explored in 10 studies [23–25, 51, 54, 57, 58, 60, 62, 63]. Two articles [13, 56] compared the remnants of soft tissue, and other 6 articles [22, 52, 53, 55, 59, 61] studied the disinfection efficacy. In terms of experimental subjects, 16 studies [13, 22, 23, 25, 50–58, 60, 61, 63] were carried out using extracted human permanent teeth, and 3 studies [24, 59, 62] used canal models. The oscillation frequency of UAI in most included studies was set in the 28–40 kHz range, while ED was driven at a frequency of 5 kHz or 6 kHz, different from UAI at a factor of 4.7–6.7. Only 1 article [57] analyzed the activation effects after canal instrumentation and during instrumentation, while the other 18 studies explored only the former.

**Table 2:** Characteristics of the included studies exploring the removal of smear layer, debris and soft tissue

Study (year)	Sample size per group	Sample types	Apical preparation size	Parameters of UAI tips	Power of ED	Placement of tips	Volume and concentration of activatied irrigants and activation time	Research indications	Obser-vational sites	Evaluation methods	Main results
Al-Rujaib et al. (2022) [63]	20	Single canals of lower premolars	40 / 06	25 / 00 30 kHz	6 kHz	WL-2 mm	10 ml 5.25%NaOCl 5 ml 17%EDTA 60 s × 3	Smear layer and debris	Coronal Middle Apical	SEM analysis 400 × magnifi-cation for debris 1000 × for smear layer 5-grade scoring system	Significant-ly less debris at apical thirds and less smear layer at all thirds in ED than UAI
Haupt et al. (2020) [49]	20	Curved (20°-40°) mesiobuccal canals of lower molars	40 / 04	15 / 02 30 kHz	5 kHz	WL-1 mm	6 ml 5%NaOCl 20 s × 3	Smear layer and debris	Coronal Apical	SEM analysis 200 × magnifi-cation for debris 1000 × for smear layer 5-grade scoring system	No significant differences between UAI and ED
Plotino et al. (2021) [52]	10	Straight (< 10°) single round canals of single-rooted teeth	40 / 06	15 / 00 30 kHz	5 kHz	WL-1 mm	3 ml 5%NaOCl 20 s × 3 Exp 1: activated after instrumen-tation Exp 2: activated during and after instrumentation	Smear layer and debris	Coronal Middle Apical	SEM analysis 1000 × magnifi-cation for smear layer and debris 4-grade scoring system	No significant differences between UAI and ED
Urban et al. (2017) [55]	12	Single round canals of lower premolars	40 / 06	15 / 00 30 kHz	6 kHz	WL-1 mm	3%NaOCl 30 s × 3	Smear layer and debris	Coronal Middle Apical	SEM analysis 200 × magnifi-cation for debris 1000 × for smear layer 5-grade scoring system	No significant differences between UAI and ED
Zhang et al. (2021) [45]	15	Straight (< 10°) single canals of lower premolars	40 / 06	25 / 02 NM	6 kHz	WL-2 mm	3 ml 17%EDTA 3 ml 3%NaOCl 30 s × 2	Smear layer	Coronal Middle Apical	SEM analysis 1000 × magnifi-cation for smear layer 5-grade scoring system	No significant differences between UAI and ED
Linden et al. (2020) [22]	9	Curved mesial canals connected by an isthmus of lower molars	30 / 07	20 / 00 45% of the maximum power (‘yellow 9’)	6 kHz	WL-2 mm	3 ml 2.5%NaOCl 20 s × 3	Debris	Canals and isthmus	Percentage of debris reduction evaluated by micro-CT voxel size of 12 µm	Significant-ly more debris removed by UAI than ED
Rödig et al. (2019)[24]	10	Curved (10°-25°) Vertucci II mesial canals of lower molars	25 / 08	25 / 00 30 kHz	6 kHz	WL-2 mm	5 ml 1%NaOCl 2 ml 17%EDTA 20 s × 4	Debris	Canals and isthmus	Percentage of debris reduction evaluated by micro-CT voxel size of 10.5 µm	No significant differences between UAI and ED
Rodrigues et al. (2021)[53]	8	Curved (20°-46°) Vertucci I mesial canals of lower molars	25 / 08	15 / 02 medium power	6 kHz	UAI: WL-2 mm ED: WL-1 mm	10 ml 5%NaOCl 5 ml 17%EDTA 20 s × 3	Debris	Canals and isthmus	Percentage of debris reduction evaluated by micro-CT voxel size of 12.8 µm	No significant differences between UAI and ED
Alsubait et al. (2021) [46]	14	Curved (10°-25°) mesial canals with isthmus of lower molars	30 / 09	20 / 00 power 8	6 kHz	WL-1 mm	2 ml NM NaOCl 30 s × 3	Debris	3 mm and 5 mm from the apex	Percentage of debris reduction evaluated by a stereomicroscope at 50 × magnifi-cation	No significant differences between UAI and ED
Al-Jadaa et al. (2023) [62]	9	Resin blocks with 2 canals connected by an isthmus containing artificial debris	45 / 05	25 / NM 38 kHz	6 kHz	2 mm from the apical foramen	1 ml 1.3%NaOCl 20 s × 3	Debtis	Isthmus	Cleared surface area in the isthmus recorded by a camera	No significant differences between UAI and ED
Plotino et al. (2019) [23]	Tests repeated 10 times per group	Canal resin models with 3 circular cavities	2.5 mm / 00^a^	Group 1: 15 / 02 40 kHz Group 2: 15 / 02 28-36 kHz Group 3: 15 / 00 28-36 kHz	6 kHz	WL-1 mm	Exp 1: NM 5%NaOCl 20 s × 3 Exp 2: NM 17%EDTA 20 s × 3	Debris	Coronal, middle and apical semi-circles	Percentage of debris reduction evaluated by a digital camera	Significant-ly more debris removed by ED than all UAI groups
Conde et al. (2017) [12]	10	Maxillary central incisors	30 / 06	20 / 00 power 4	NM	WL-2 mm	3 ml 2.5%NaOCl 1 ml 17%EDTA 30 s × 2	Soft tissue	Artificial grooves at WL-2 mm and WL-6 mm	Percentage of weight reduction of pigs' palatal mucosa in the grooves	No significant differences between UAI and ED
Iandolo et al. (2021) [51]	Tests repeated 20 times per group	An upper single-rooted premolar with two root canals and an isthmus	25 / NM	15 / 02 40 kHz	6 kHz	WL-1 mm	Exp 1: 10 ml NM NaOCl 30 s × 10 Exp 2: 10 ml NM heated NaOCl 30 s × 10	Soft tissue	Isthmus	Area of pulp tissue after irrigation recorded by a digital camera and calculated in pixels	No significant differences between UAI and ED in exp 1 or exp 2

*UAI* ultrasonically-activated irrigation, *ED* EDDY, *WL* Working length, *SEM* scanning electronic microscope, *Exp* experiment, *NM* not mentioned

^a^The resin model used in the study were 2.5 mm in width and had no taper

**Table 3:** Characteristics of the included studies exploring the removal of microbes

Study (year)	Sample size per group	Sample types	Apical preparation size	Para-meters of UAI tips	Power of ED	Placement of tips	Volume and concentration of activatied irrigants and activation time	Evaluation methods				Main results
								Bacterial species	Culture time in canals	Sampling	Examination	
Eneide et al. (2019) [47]	12	Single-rooted human teeth	25 / 06	NM	6 kHz	WL-1 mm	6 ml 5.25% NaOCl 6 ml 17%EDTA 20 s × 6	Enterococcus faecalis	28 days	A canal brush scratching canal walls	Colony forming units	No significant differences between UAI and ED
Hage et al. (2019) [48]	10	Single-rooted lower premolars	25 / 08	15 / 02 40 kHz	6 kHz	WL-1 mm	9 ml 5.25% NaOCl 30 s × 3	E. faecalis	7 days	A paper point obtaining liquid	Colony forming units	No significant differences between UAI and ED
Hoedke et al. (2021) [50]	20	Straight canals of upper anterior teeth	Exp 1: infection in 25 / 06 canals and subsequent preparation to 40 / 06 Exp 2: 40 / 06	25 / 00 30 kHz	6 kHz	WL-1 mm	Protocol 1: 10 ml 0.9% NaCl 30 s × 2 Protocol 2: 10 ml 1% NaOCl 30 s × 2	E. faecalis and Streptococcus oralis	Before irrigation: 5 days After irrigation: 5 days	Sample 1: a paper point obtaining liquid Sample 2: a Hedström file scratching canal walls	Colony forming units	Exp 1: No significant differences between UAI and ED Exp 2: Signifi-cantly greater bacterial reduction by ED than UAI
Neuhaus et al. (2016) [21]	Exp 1: 5 straight (< 15°) roots and 5 curved (> 25°) roots per group Exp 2: 6	upper premolars and front teeth, and palatal roots from upper molars	25 / 08	NM 20% power	6 kHz	UAI: WL-1 mm ED: WL	Exp 1: NM 0.9% NaOCl 20 s × 3 Exp 2: NM 1.5% NaOCl 20 s × 3	1. Streptococcus gordonii 2. Actinomyces oris 3. Fusobacterium nucleatum 4. S. gordonii and A. oris 5. S. gordonii and F. nucleatum 6. E. faecalis 7. Candida albicans 8. Clinical retreatment isolates (1–3 and 8 just for Exp 1; 4–7 for both Exp 1 and 2)	Exp 1: 3 days Exp 2: 21 days	A paper point obtaining liquid	Colony forming units	Exp 1: significantly less remaining micro-organisms by ED than UAI in both straight and curved canals Exp 2: both ED and UAI were less effective against E.faecalis and C.albicans
Swimberghe et al. (2021) [54]	20	Acrylic models with curved (30° or 45°) canals	40 / 06	25 / 00 30 kHz	6 kHz	UAI: WL-2 mm ED: WL-1 mm	Water 20 s × 3	Area of the biofilm-mimicking hydrogel in the apical grooves recorded by a digital camera and calculated in pixels				30° group: No significant differences 45° group: significantly more hydrogel removed by UAI than ED
Yared et al. (2020) [56]	10	Single-rooted lower premolars	25 / 08	20 / 02 power 4	NM	WL-1 mm	Exp 1: NM 5.25%NaOCl at room temperature 20 s × 3 Exp 2: NM 5.25%NaOCl heated by 150℃ heat carrier 20 s × 3	E. faecalis	28 days	#15 hand file scratching canal walls, then a paper point obtaining liquid	Colony forming units	No significant differences between UAI and ED in exp 1 or exp 2

*UAI* ultrasonically-activated irrigation, *ED* EDDY, *WL* Working length, *Exp* experiment, *NM* not mentioned

### Description of Different Indicators

#### Smear layer

The effects on smear layer removal of UAI and ED were all evaluated with SEM in the 5 relevant studies. No significant differences were observed between the two activation methods in 4 studies [50, 54, 57, 60], whereas Al-Rujaib et al. [63] found significantly more smear layer was removed by ED than UAI at coronal, middle and apical thirds. Plotino et al. [57] suggested no significant differences in the removal of the smear layer when UAI or ED was used both during and after instrumentation.

#### Debris

Four studies [54, 57, 60, 63] evaluated the debris remnants via SEM. Other 3 studies [23, 25, 58] compared the debris reduction in curved canals with isthmus using micro-CT. Alsubait et al. [51] explored the debris reduction at the cross section of the canal isthmus observed by a stereomicroscope. Plotino et al. [24] and Al-Jadaa et al. [62]. used canal resin blocks filled with dentin debris to assess debris removal efficacy by the digital camera. Among all these 10 studies, Al-Rujaib et al. [63] observed significantly less debris left at the apical thirds after ED irrigation than UAI under SEM, while Linden et al. [23] found significantly more debris removed by UAI than ED using micro-CT analysis; the rest 8 studies found no significant differences between them.

#### Soft tissue

Conde et al. [13] used pigs’ palatal mucosa to mimic pulp tissue and found no significant differences in soft tissue reduction after UAI or EDDY activation. Iandolo et al. [56] conducted all the experiments with pulp tissue from premolars and showed comparable results between UAI and ED. No meta-analysis was performed due to the different methodologies of the 2 studies.

#### Disinfection

Five articles [22, 52, 53, 55, 61] inoculated canals with microbes and counted colony-forming units to explore the disinfection ability of ED compared to UAI. Inoculated bacteria included Enterococcus faecalis [22, 52, 53, 55, 61], Streptococcus [22, 55], Actinomyces viscosus [22], Fusobacterium nucleatum [22], Candida albicans [22] and intracanal isolates from endodontic retreatment [22]. Two studies [22, 55] indicated that ED was more effective against microbes than UAI in root canals contaminated after instrumentation. The rest 3 articles [52, 53, 61] found no significant differences in bacterial elimination between UAI and ED. On the other hand, Swimberghe et al. [59] used biofilm-mimicking hydrogel and found that UAI removed significantly more hydrogel than ED. No meta-analysis was conducted on disinfection efficacy due to the different methods and reported outcomes of the included studies.

#### Meta-analysis

**Fig. 2:**
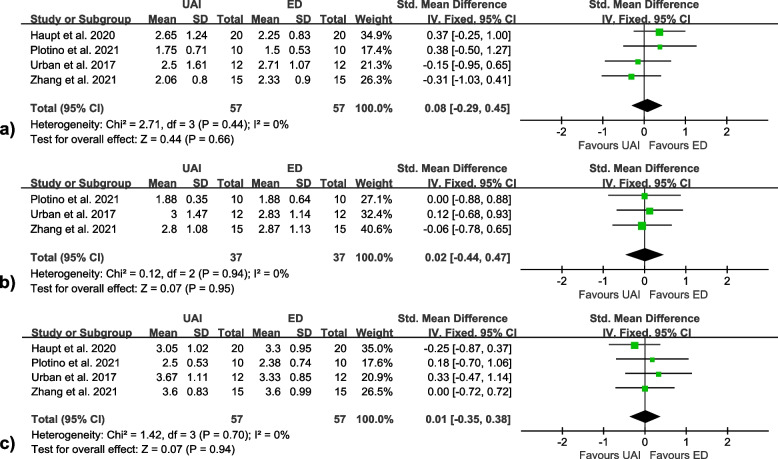
Forest plots of smear layer removal at **a** coronal, **b** middle and **c** apical thirds

**Fig. 3:**
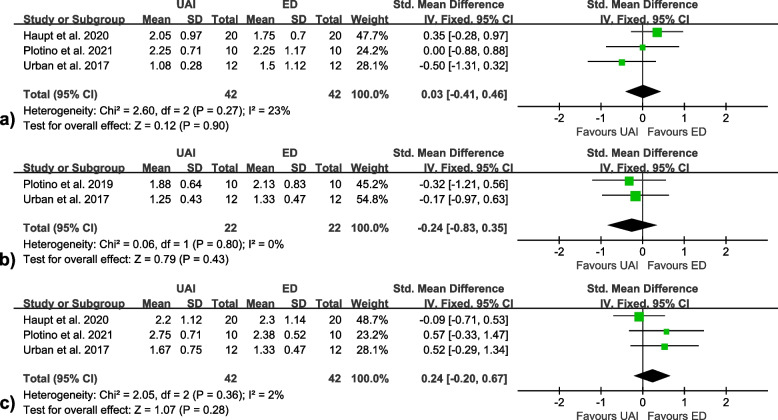
Forest plots of debris removal at **a** coronal, **b** middle and **c** apical thirds evaluated by SEM

**Fig. 4:**
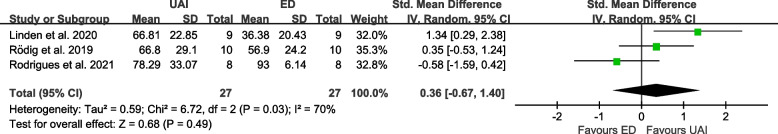
Forest plot of debris removal evaluated by micro-CT

Four studies [50, 54, 57, 60] were included in the meta-analyses on the smear layer removal, and no significant differences were found between UAI and ED at all canal thirds (coronal [SMD = 0.08, 95% CI: -0.29 to 0.45, I^2^ = 0%]; middle [SMD = 0.02, 95% CI: -0.44 to 0.47, I^2^ = 0%]; apical [SMD = 0.01, 95%CI: -0.35 to 0.38, I^2^ = 0%]) (Fig. 2). Meta-analyses on the debris removal were conducted separately on the 3 SEM studies [54, 57, 60] and the 3 micro-CT studies [23, 25, 58]. Both the former (coronal [SMD = 0.03, 95% CI: -0.41 to 0.46, I^2^ = 23%]; middle [SMD = -0.24, 95% CI: -0.83 to 0.35, I^2^ = 0%]; apical [SMD = 0.24, 95%CI: -0.20 to 0.67, I^2^ = 2%]) (Fig. 3) and the latter (SMD = 0.36, 95% CI: -0.67 to 1.40, I^2^ = 70%) (Fig. 4) found no significant differences between the two activation methods.

#### Quality assessment

**Fig. 5:**
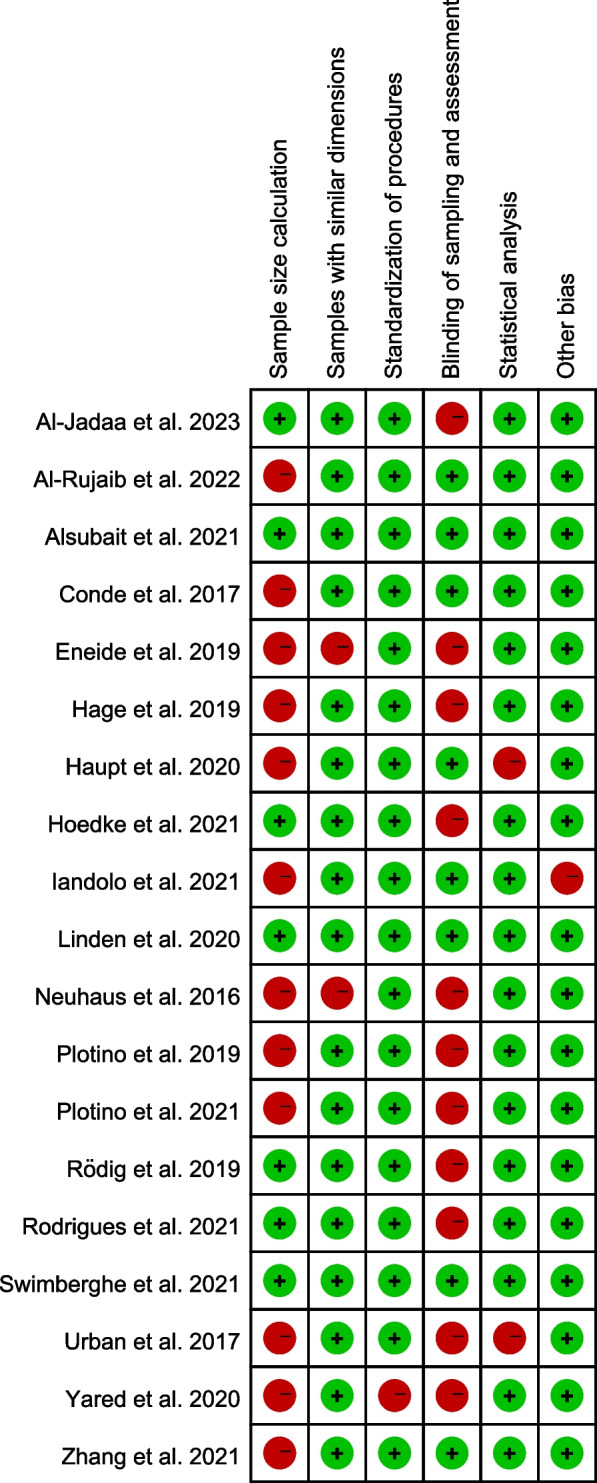
Risk of bias of each included study

**Fig. 6:**
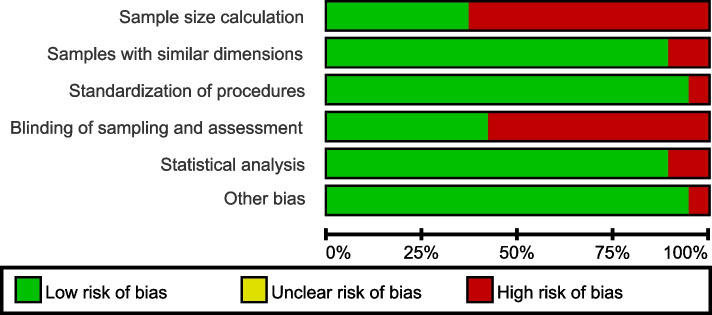
Summary of risk of bias

The quality assessments on all the 19 eligible studies were listed in Figs. 5 and 6. The overall risk of bias in the included studies was evaluated as low (*n* = 3) [23, 51, 59], moderate (*n* = 12) [13, 24, 25, 50, 53–58, 62, 63] and high (*n* = 4) [22, 52, 60, 61]. Most studies did not conduct sample size calculation or blinding during sampling and outcome assessment.

## Discussion

This systematic review aimed to compare the cleaning efficacy of the EDDY activation system with ultrasonically-activated irrigation in root canals. We excluded the studies without closed canal systems. Root apices are naturally surrounded by periodontium, which can act as a barrier to the overflow of rinsing fluid during clinical treatments. Sealed apical foramens of extracted teeth and canal models can mimic the in vivo environment better than open ones. Tay et al. [64] found that closed canal systems achieved significantly worse cleanliness in canals than the open ones after irrigation. This might be attributed to intracanal matter flushing out through the apical foramen in the open-end system. However, it was worth noting that one-third of the excluded articles were eliminated owing to not sealing apices. More attention should be paid to the importance of apical disclosure in irrigation experiments.

The ability of ED and UAI to remove the smear layer was studied in 5 articles [50, 54, 57, 60, 63]. Four of them [50, 54, 57, 60] found no significant differences between the two activation systems at all canal thirds. These results are consistent with the outcomes of the meta-analysis on the smear layer with unimportant heterogeneity. Only Al-Rujaib et al. [63] found ED significantly better than UAI at all canal portions, but the experimental data were unobtainable, and this article was thus not included in meta-analyses. The results of the 5 studies indicated that the ability of ED to remove the smear layer was at least comparable to that of UAI, but further investigations are needed to prove the superiority of ED. Paixão et al. [65] conducted a meta-analysis comparing the smear layer removal between ultrasonically-activated irrigation and sonically-activated irrigation. The analysis found that UAI had significantly poorer performance than the latter at apical thirds with substantial heterogeneity. Nevertheless, this quantitative synthesis included only 2 articles, which applied ED and the EndoActivator activation system (33-167 Hz; Dentsply, Tulsa, OK) as the final sonic activation systems. Although both systems vibrate at acoustic frequencies, their frequencies differ by more than 30 times. So wide is the gap that it might lead to different cleaning effectiveness, which could be one of the sources of the substantial heterogeneity. Therefore, the significance of this meta-analysis remained to be discussed.

Totally 10 studies [23–25, 51, 54, 57, 58, 60, 62, 63] compared the debris elimination between UAI and ED using various methods. Plotino et al. [24] found ED removed significantly more debris than UAI using canal resin models filled with dentinal debris. Nevertheless, the simulated main canals and accessory canals in this study were much wider than the actual ones in human teeth. Thus, the experimental results might deviate from clinical practice. Al-Jadaa et al. [62] also conducted experiments on resin blocks but with a much more realistic canal system, and detected similar effects between ED and UAI in the closed canal system. Alsubait et al. [51] observed debris in isthmuses under a stereomicroscope at 50 × magnification and found no significant differences between the two activation methods. 3 studies [23, 25, 58] evaluated debris in curved canals connected by isthmuses using micro-CT. Their quantitative synthesis showed insignificant differences between UAI and ED but with substantial heterogeneity. This heterogeneity might arise from different canal instrumentation systems, preparation sizes and parameters of UAI. The absence of 17%EDTA in the final irrigation protocol [23] might also be accountable for the heterogeneity, as 17%EDTA was effective in debris elimination [6]. Other 4 studies [54, 57, 60, 63] conducted SEM analysis to assess debris remnants, and their meta-analyses (except Al-Rujaib et al. [63] as mentioned above) found ED as effective as UAI at any canal third with unimportant heterogeneity.

Devices like digital cameras or stereoscopes allow for rough observation of canal walls but can hardly discover tiny chips due to their low resolution. In this regard, scanning electron microscopes and micro-CT with high resolution can perform better. However, it is undeniable that SEM and micro-CT have their limitations when applied to canal irrigation experiments. Longitudinal observation for pre- and post-irrigation comparison is impracticable in SEM analysis [66] due to the necessary process of dehydration and metallization. Orlowski et al. [67] evaluated the smear layer before the final irrigation using low-vacuum SEM after only dehydration without gold sputtered, and observed the same areas after irrigation under high-vacuum SEM. Although this method allowed for longitudinal evaluation, the process of desiccation may alter the structure of smear layer, which contains water-bearing soft tissue, biofilms and dentinal debris [68]. Desiccation may lead to greater brittleness [69] of smear layer and debris and makes them more removable. Given the drawbacks of SEM, micro-CT was recommended as it was capable of non-destructive three-dimensional imaging and allowed for longitudinal observation [6]. However, micro-CT omits debris with low radiopacity such as soft-tissue chips and biofilms that are distinguishable under SEM [70]. From this perspective, it can be deduced that although each observational method has its defects, the overall consideration of all the results from different methods may offset their weakness and improve the reliability. Consequently, in view of all the results and factors mentioned above, a conclusion can be drawn that ED is comparable to UAI in removing the smear layer and debris.

The efficacy of soft tissue removal was studied in only 2 included articles. Conde et al. [13] found ED was as effective as UAI in eliminating soft tissue placed in artificial grooves by weighing the tissue pre- and post-irrigation. Iandolo et al. [56] calculated the area of the pulps placed in the isthmus and also found no significant differences. These results suggested that ED might have similar effects to UAI on removing soft tissue in complex anatomic sites in root canals.

Six studies explored the disinfection effects. Neuhaus et al. [22] introduced different species of microbes into both straight and curved root canals. The results showed that EDDY was significantly more effective than that of UAI in all the short-term disinfection experiments. But these results were based on the experiments conducted with only normal saline as the final irrigant, which might greatly reduce the disinfection efficacy. Moreover, EDDY tips were placed at the working length, which was different from the depth of UAI tips and might cause severer apical extrusion in clinical practice [71]. Thus, the clinical extrapolation of the results should be done with caution. Hoedke et al. [55] found ED significantly better than UAI at disinfection when the canals were contaminated after the entire instrumentation process. But it also found no significant difference when the contamination was done before the instrumentation of the final file. This contradiction might be attributed to the partial removal of bacteria during the mechanical preparation. The gap between ED and UAI in disinfection ability might thus be narrowed down to insignificance. However, the above-mentioned two experiments were short-term (3 and 5 days respectively) infection models, where the microbes had not yet penetrated deeply into dentinal tubules [72, 73]. In long-term (28 days) infection models [52, 61], ED and UAI showed comparable results in eliminating Enterococcus faecalis. On the other hand, Swimberghe et al. [59] used hydrogel to mimic pulp tissue in complex anatomic sites. This study found that UAI removed significantly more hydrogel than ED in canal models with a curvature of 45 degrees. Although the hydrogel mixture was demonstrated to share similar viscoelastic behaviors to biofilms, whether the shear stress required to remove the hydrogel was similar to that of biofilms remains unknown. Shear stress also plays a role in microbe elimination [74]. Furthermore, disinfection not only lies on the smash and removal of biofilms by mechanical washing, but also counts on sufficient contact of antimicrobial irrigants with intracanal microbes to disable their toxicity and fertility. To sum up, despite the different experimental designs and outcomes, it can be inferred that ED was as effective as UAI in disinfection.

The oscillation frequency of UAI is more than four times higher than that of ED. UAI can generate cavitation and acoustic streaming in water with its high-frequency vibration [16]. But no cavitation was detected during EDDY activation [45]. And ED might not be able to produce acoustic streaming due to its high amplitude (approximately 350 μm [45, 75]) according to the theoretical analysis [76]. However, despite the inability to generate cavitation and acoustic streaming, ED seemed to achieve comparable cleaning efficacy as UAI did according to the results in the present review. This implied that oscillation frequency might not be the most crucial factor for root canal irrigation. ED has a higher amplitude than UAI [45]. EDDY tips make three-dimensional orbital movements [45], while UAI files oscillate transversely in one plane [77]. These facts suggested that the amplitude or the oscillation direction might also play an important role in root canal irrigation. But more basic researches are needed to further explore the mechanism of ED for irrigation activation. On the other hand, too high a frequency of UAI might cause damage to canal walls. Al-Jadaa et al. [78] applied irrigation to resin blocks and found that ultrasonic stainless steel tips produced canal ledgings and transportation while polymer tips of sonic activation did not. Experiments on extracted teeth also detected unintentional removal of dentin after UAI in both straight [79] and curved [80] canals. It could be inferred from these findings that UAI assisted in removing the smear layer and debris but meanwhile probably produced them. In addition, although the high frequency of UAI led to a greater increase in flow rate and changed liquid from laminar to turbulent flow, the laminar was more conducive to irrigants flowing into narrow anatomic sites because of its regularity [81]. This could be one of the reasons why most included articles that explored cleaning efficacy in isthmus found no superiority of UAI over ED. Another difference between UAI and ED was that the former could result in a higher temperature rise in sodium hypochlorite solution than the latter [82]. But this small temperature difference (< 10 ℃) caused by activation was insufficient to enhance the reaction rate of NaOCl [83]. However, the temperature rise by more than 15 ℃ in the solution could reduce viscosity and increase mobility [81]. This change might improve the ability to eliminate soft tissue [56] and microbes [61]. The combination of activation and heating of irrigants could therefore enhance the effects of root canal irrigation.

Although the frequency of EDDY is within the range of sonic vibration, the results in the present review cannot be extended to other sonically-activated systems, as they are widely different in the oscillation frequency as mentioned above, and even diverse in the operating modes [84–87].

The studies included in the present review varied widely in experimental methods and outcome measures, especially in the aspects of debris and soft-tissue removal and disinfection, which greatly limited the availability of meta-analyses. Also, the small sample size of each dimension might impair the reliability of this research. Another limitation of this review is that the effectiveness of root canal irrigation was evaluated from an in vitro perspective. The in vitro environments differed from the in vivo ones considerably. Patient factors such as tooth position, mouth opening and systemic diseases might affect the application of UAI and ED, and thus might result in different irrigation effects from that of in vitro experiments. Besides, most included studies decorated the extracted teeth to standardize the sample length, which deviated from clinical practice because the normal coronal approach of working tips was altered. Therefore, caution must be taken in the interpretation and the clinical extrapolation of the results in this review.

## Conclusions

After the qualitative and quantitative analysis of the included articles, it could be concluded from the limited evidence that ED was at least equivalent to UAI in root canal irrigation concerning the removal of smear layer, debris, soft tissue and bacteria ex vivo. Considering the metal tips of UAI may damage canal walls, EDDY might be a substitute for UAI to activate irrigation solutions. However, owing to the different circumstances between in vitro experiments and clinical practice, more randomized clinical trials are required to explore the clinical extrapolation of the conclusion in the present review.

## Data Availability

All data generated or analyzed during this study are included in the present review.

## Abbreviations

UAI: Ultrasonically-activated irrigation
ED: EDDY activation system
SMD: Standardized mean difference
SEM: Scanning electron microscope
CI: Confidence interval
WL: Working length
Exp: Experiment; NM: not mentioned
